# IBC CARe Microarray Allelic Population Prevalences in an American Indian Population

**DOI:** 10.1371/journal.pone.0075080

**Published:** 2013-09-06

**Authors:** Lyle G. Best, Cindy M. Anderson, Richa Saxena, Berta Almoguera, Hareesh Chandrupatla, Candelaria Martin, Gilbert Falcon, Kylie Keplin, Nichole Pearson, Brendan J. Keating

**Affiliations:** 1 Science Department, Turtle Mountain Community College, Belcourt, North Dakota, United States of America; 2 School of Medicine and Health Sciences, University of North Dakota, Grand Forks, North Dakota, United States of America; 3 College of Nursing, University of North Dakota, Grand Forks, North Dakota, United States of America; 4 Center for Human Genetics Research, Massachusetts General Hospital, Harvard Medical School, Boston, Massachusetts, United States of America; 5 Centre for Applied Genomics, The Children’s Hospital of Philadelphia, Philadelphia, Pennsylvania, United States of America; New Jersey Institute of Technology, United States of America

## Abstract

**Background:**

The prevalence of variant alleles among single nucleotide polymorphisms (SNPs) is not well known for many minority populations. These population allele frequencies (PAFs) are necessary to guide genetic epidemiology studies and to understand the population specific contribution of these variants to disease risk. Large differences in PAF among certain functional groups of genes could also indicate possible selection pressure or founder effects of interest. The 50K SNP, custom genotyping microarray (CARe) was developed, focusing on about 2,000 candidate genes and pathways with demonstrated pathophysiologic influence on cardiovascular disease (CVD).

**Methods:**

The CARe microarray was used to genotype 216 unaffected controls in a study of pre-eclampsia among a Northern Plains, American Indian tribe. The allelic prevalences of 34,240 SNPs suitable for analysis, were determined and compared with corresponding HapMap prevalences for the Caucasian population. Further analysis was conducted to compare the frequency of statistically different prevalences among functionally related SNPs, as determined by the DAVID Bioinformatics Resource.

**Results:**

Of the SNPs with PAFs in both datasets, 9.8%,37.2% and 47.1% showed allele frequencies among the American Indian population greater than, less than and either greater or less than (respectively) the HapMap Caucasian population. The 2,547 genes were divided into 53 functional groups using the highest stringency criteria. While none of these groups reached the Bonferroni corrected p value of 0.00094, there were 7 of these 53 groups with significantly more or less differing PAFs, each with a probability of less than 0.05 and an overall probability of 0.0046.

**Conclusion:**

In comparison to the HapMap Caucasian population, there are substantial differences in the prevalence among an American Indian community of SNPs related to CVD. Certain functional groups of genes and related SNPs show possible evidence of selection pressure or founder effects.

## Introduction

Substantial progress in understanding the genetic contribution to human variation and disease has been made in the past few decades; but the practical application of this knowledge has not met (perhaps exaggerated) expectations [1]. In particular, the increased relative risk of genetic variants for adverse medical conditions in specific populations has been determined through many case/control and population-based cohorts, using candidate gene and genome-wide association (GWA) studies. The population attributable risk, however, of these variants is highly dependent on their allele frequency, in concert with interacting genetic and environmental covariates unique to that population. The population prevalence of these alleles is also critical to the design of further genetic epidemiology studies and consideration of public health screening measures. Most of the current data on population allele frequency (PAF) is derived from relatively small cohorts, focused on major, self-reported ethnic groups, such as the HapMap [2,3] dataset. Larger cohorts have been genotyped for small numbers of genetic variants, again, primarily among the major ethnicities [4–6]. A consortium comprised of the Institute of Translational Medicine and Therapeutics, the Broad Institute and the Candidate Gene-Association Resource (CARe) led the development of a 50K SNP, custom genotyping microarray (the “IBC array”). This array focuses on candidate genes and pathways with demonstrated influence on inflammatory pathways, metabolic phenotypes and the pathophysiology of cardiovascular disease (CVD). The present study utilizes genotypes from the IBC CARe microarray obtained from the control arm (N=216) of a pre-eclampsia study among an American Indian population in the northern plains of the United States. Population prevalences were determined and compared with HapMap Caucasian and other PAFs. Bioinformatics software was then used to parse the SNPs into functionally related groups and these groups were searched for functionalities that exhibit significant group differences in PAF.

## Methods

Ethics Statement: Approval was obtained from both the Aberdeen Area Indian Health Service (IHS) and University of North Dakota Institutional Review Boards and the tribal government. Individual, written informed consent was obtained from each participant. The data raw cannot be deposited to a public repository, in accordance with the requirements of our Institutional Review Board and in order to protect participant privacy. However, this data will be made available upon request to qualified researchers for analyses consistent with our consents.

The basis for this analysis are genotypes solely from controls recruited for a Turtle Mountain Community College (TMCC) study of pre-eclampsia between 8/04 and 7/12 [7]. The federally funded IHS, through the hospital and clinic located in Belcourt, North Dakota, is the primary health care provider for eligible tribal members of the Turtle Mountain Band of Chippewa. Controls were identified by automated query of an electronic medical record database (the Resource, Patient, Management System [RPMS]) at this facility, using a relevant group of ICD9 codes. Two controls were ascertained by contact of the first individual to deliver before and after the index case. If a potential control declined participation, the woman delivering during the next prior or subsequent day was contacted; and this was continued until two controls were recruited. This method of recruiting controls provides a relatively unbiased, population-based sample.

Template DNA was collected and processed using salivary samples and the Oragene (DNA Genotek Inc) system. Genotyping of the anonymized samples was accomplished by microarray analysis on the IBC (version 1) microarray at The Children’s Hospital of Philadelphia. SNPs genotyped are listed at the IBC Array website [8]. Quality control standards were monitored with the mean call rate above 98% for all SNPs on the microarray and less than 4% of samples had a SNP call rate below 95%.

Results of the microarray genotyping will be available to qualified investigators with assurances that 1) no attempts will be made to identify individuals, 2) goals of the analysis are within the scope of the consent and not for anthropologic research, and 3) the results will not be used for commercial purposes.

During initial data analysis a correlation was noted between the PAF of a subset (13.1%) of SNPs and (100 – PAF) of the comparison population. This was persisted after careful attention to possible inconsistencies between strand designation for the IBC microarray and the NCBI standard reference allele. Strand conversion was suspected as the cause and elimination of all SNPs consisting of GC and/or AT pairs corrected this situation.

HapMap (phases II + III) Caucasian (CEU) and Han Chinese, Denver (CHD) autosomal allele frequencies of the IBC SNPs were obtained from the HapMap website [9]. Allele frequencies for the present study were calculated by counting all available genotypes for a SNP and 95% confidence intervals were calculated as the mean PAF +/- 1.96* (√((PAF)*(1-PAF)/(total number of alleles)) to give the upper and lower bounds. For PAFs at the margins of the distribution (i.e. 0-1% or 99-100%), the normal approximation to the binomial distribution is not applicable; as irrational results, such as a lower 95% CI of 100% for a PAF of 100% are obtained. For this reason, the upper confidence intervals were set at 1% for a mean of 0% and the lower confidence interval 99% when the mean was 100%. The 1% value was arrived at by observing that typical estimated PAFs of 99% had lower 95% CIs about 1% less than the mean.

Allele frequencies from two populations were designated significantly different if the upper and lower 95% confidence intervals did not overlap. The median HapMap sample size was 112 individuals or 224 alleles (mean = 186 alleles), whereas the median sample size was 216, or 432 alleles (mean = 424 alleles) for the TMCC cohort.

To assess the potential impact of non-Indian admixture on these findings, Multidimensional Scaling (MDS) analysis was performed using PLINK *v 1.07* [10]. At the SNP level, quality control was performed and SNPs with genotype call rate ≥0.95, a minor allele frequency ≥0.1, and a p-value from the Hardy-Weinberg equilibrium test ≥10^-6^ were kept. A composite score of the three principal components from this analysis was used to dichotomize the population into those most closely related to CEU and those least related to CEU, which is the ethnic group in closest genetic proximity to this American Indian population. The proportion of SNPs with statistically significant PAFs was then determined for these two groups in the same fashion as described above. See Figure S1 for a graphic representation.

The National Institute of Allergy and Infectious Disease bioinformatic website, Database for Annotation, Visualization and Integrated Discovery (DAVID) v 6.7 [11,12], operating under the “highest” classification stringency, was used to categorize the functionality of the 34,240 TMCC SNPs into 53 groups. Within these groups, the number of SNPs with statistically significant differences in PAF was analyzed using the Excel binomial distribution function and the average number of SNPs with differing PAFs among those included in the groups (47.1%). The probability of finding a group with this number of differing PAFs was evaluated considering the Bonferroni correction for multiple tests, which gives a nominal p value of 0.05/53 = 0.000943. The most typical function of those groups with fewer or more differing PAFs was determined with assistance from the GeneCards [13] website and search for common terms in the “aliases and descriptions” notation for each gene.

Statistical analysis was primarily carried out using SPSS version 10.1.0 software. Descriptive statistics report mean (+/- SD) for continuous variables and proportions with 95% CI for discrete variables. Statistical significance was set at p<0.05.

## Results

**Figure 1:**
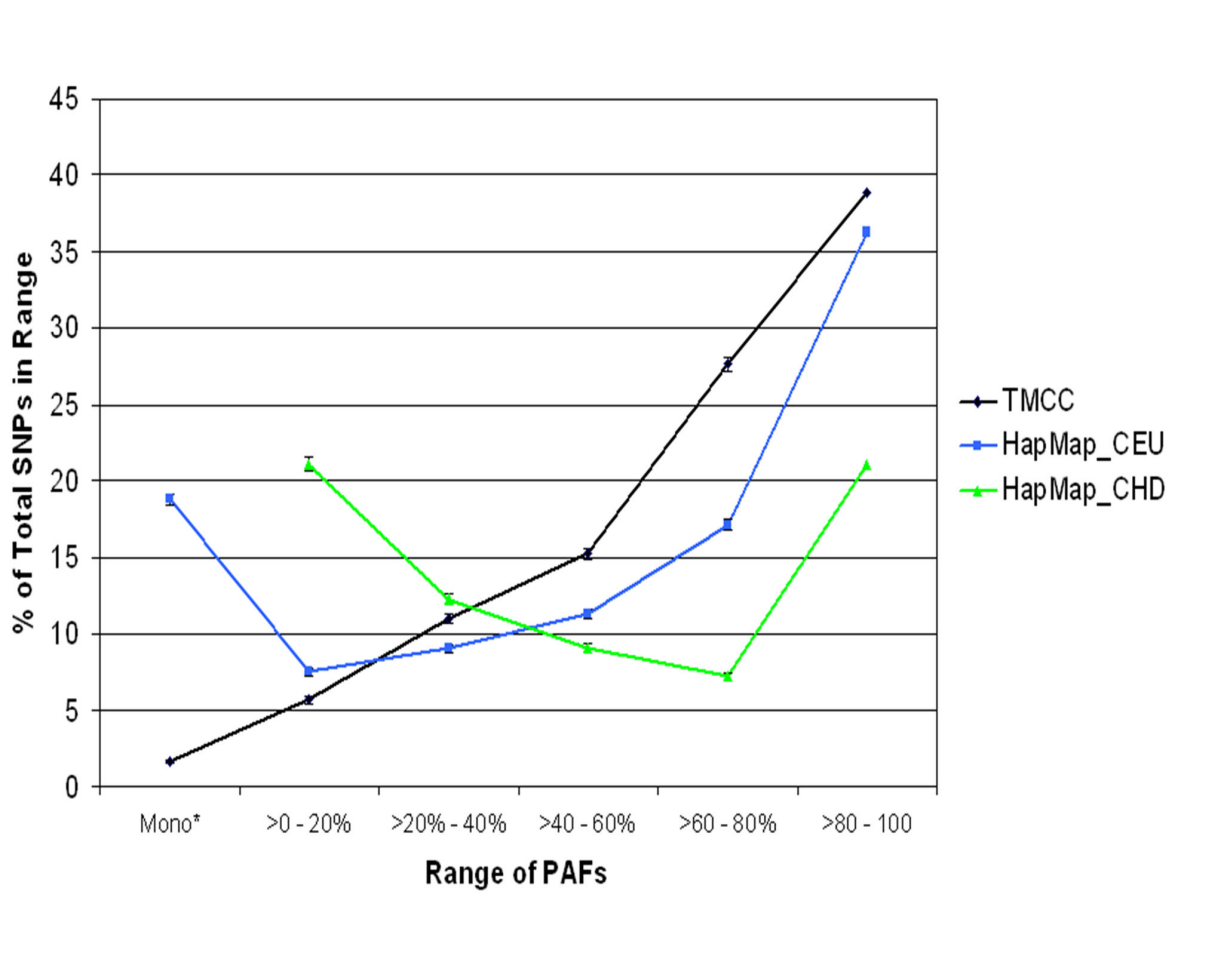
The proportion of PAFs in ranges for TMCC and HapMap populations.

Demographic characteristics of the 216 females genotyped include means (SD) of 23.75 (5.47) years, 27.56 (6.72) Kg/m^2^, 12.24 (2.21) years of age, body mass index (BMI) and education respectively. The proportion of current smokers was 46.5% (38.4%-54.7%). The HapMap CEU sample is from Utah residents with Northern and Western European ancestry; and the HapMap CHD population is from Chinese residents in metropolitan Denver, Colorado. The proportion of PAFs in various ranges in this American Indian population is shown in Figure 1.

**Figure 2:**
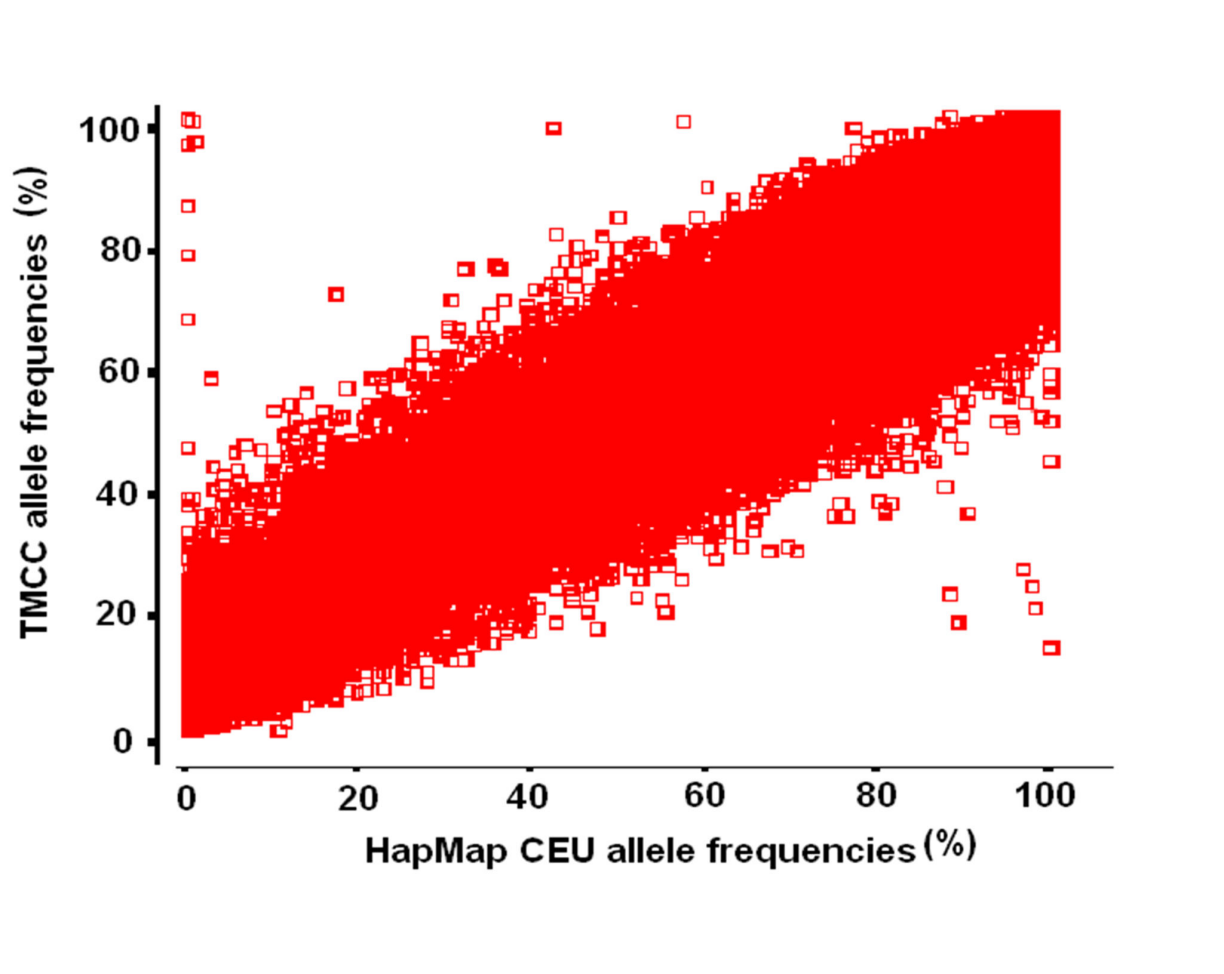
Plot of TMCC vs HapMap CEU PAFs.

**Figure 3:**
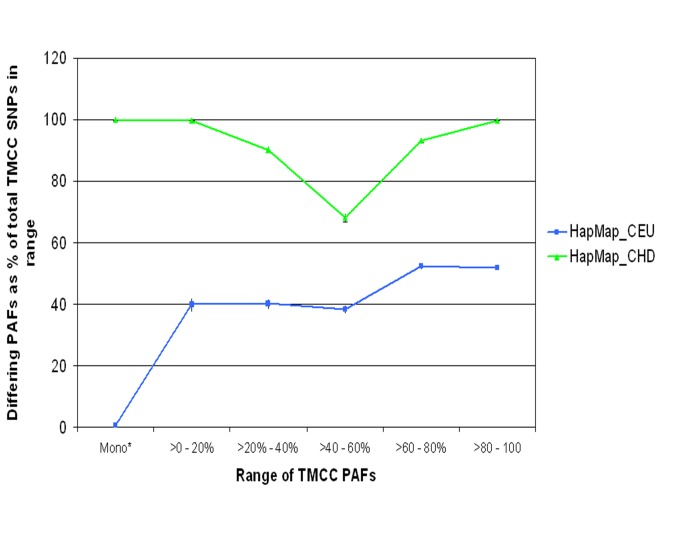
The number of SNPs in each TMCC prevalence range with a statistically significant different PAF than HapMap CEU or CHD MAFs.

A plot of PAFs from the HapMap CEU population vs the TMCC population is seen in Figure 2. Of the 34,240 SNPs that could be compared with the HapMap CEU prevalence, allele frequencies among this American Indian population were respectively 9.8%, 37.2% and 47.1% significantly greater, lesser or either. The analogous results for a comparison of Denver Han Chinese with this cohort shows 65.0%, 24.4% and 89.4% significantly greater, lesser or either. The distribution of differing PAFs between these three populations, stratified by range of American Indian PAFs, is given in Figure 3. In the range of TMCC PAFs between 20 and 80%, there were 18,451 SNPs that could be compared with HapMap CEU and 54.7% and 16.4% showed absolute differences of 10% and 20%, respectively.

To evaluate the possible influence of admixture in this population, comparisons were made between the two partitioned halves of the American Indian population (more and less closely related to Europeans) and the HapMap CEU population. This analysis resulted in lessening of the proportion of differing PAFs (partly related to loss of power from reduced population size), such that 12.4% of those most closely related to Europeans had statistically different PAFs, compared with 23.9% of those less closely related.

Comparisons between the three other reports of population-based PAFs among those of European ethnicity and this American Indian cohort are shown in Table 1. Of the 92 PAFs that could be compared between these populations and the TMCC cohort, a total of 71 (77.1%) were significantly different; and even 4 of the 7 SNPs (57%) that allowed pair-wise comparisons among 3 European cohorts themselves showed a significant difference (Table 1).

**Table 1:** Comparisons between TMCC and European PAFs reported in the literature.

NHANES [4] vs TMCC								
PAF range	0%	>0 = 20%	>20 = 40%	>40 = 60%	>60 = 80%	>80 = 100%	100%	Total
Total SNPs	1	3	1	10	14	9	0	38
Differing	0	1	1	7	12	7	0	28
% of Total	0.0	33.0	100.0	70.0	85.7	77.7	0.0	73.7
Cross et al. [6] (European) vs TMCC								
Total SNPs	0	1	4	11	8	3	0	27
Differing	0	1	2	9	6	3	0	21
% of Total	0.0	100.0	50.0	81.8	75.0	100.0	0.0	77.7
Cross et al. [6] (American Indian) vs TMCC								
Total SNPs	0	1	4	11	8	3	0	27
Differing	0	1	4	7	5	2	0	19
% of Total	0.0	100.0	100.0	63.6	62.5	66.6	0.0	70.3
Huang et al. [5] vs TMCC								
Total SNPs	0	2	3	5	13	4	0	27
Differing	0	2	2	5	9	4	0	22
% of Total	0.0	100.0	66.6	100.0	69.2	100.0	0.0	81.5
Huang et al vs Cross vs NHANES*								
Total SNPs	0	0	0	4	2	1	0	7
Differing	0	0	0	1	2	1	0	4
% of Total	0.0	0.0	0.0	25.0	100.0	100.0	0.0	57.1

* Note this last comparison is for any one of the 3 literature reports that differs from one of the others

Using the “highest” stringency criteria, there were 705 DAVID identified genes associated with 8,921 SNPs among the 53 functional groups detected. Table 2 shows these 53 groups, the number of SNPs assigned to each ranged from 2 to 972 with a mean of 168. The number of differing PAFs in each group and the binomial probability of that number (or more extreme values) are shown in Table 2. While none of the groups reach a Bonferroni corrected p value of 0.00094, in total there were 7 of these 53 groups with either more or less than expected numbers of differing PAFs. With a nominal p value of 0.05, one would expect fewer than 5 out of 53, and the finding of 7 has an overall probability of 0.0046. There were 28 of the 53 groups with more differing PAFs, compared with 25 groups with less. Assuming an equal distribution of “more” and “less” groups, this is an unremarkable distribution (p=0.392).

**Table 2:** Functional groups with significantly increased numbers of PAFs differing from HapMap CEU.

GROUP	TOTAL SNPs	DIFFERING	DAVID GENES	PROBABILITY*	DIRECTION**
2	70	38	12	0.0928	MORE
5	130	67	15	0.1353	MORE
6	100	49	12	0.3149	MORE
7	144	49	7	0.0010	LESS
8	834	378	93	0.1604	LESS
9	76	31	6	0.1618	LESS
10	84	45	9	0.0973	MORE
11	16	9	7	0.3140	MORE
12	40	17	5	0.3368	LESS
14	367	159	31	0.0810	LESS
15	65	27	6	0.2199	LESS
17	62	34	5	0.0889	MORE
18	49	25	6	0.2439	MORE
19	145	65	12	0.3215	LESS
20	94	40	7	0.2181	LESS
21	105	38	5	0.0156	LESS
22	50	24	9	0.3931	MORE
23	91	36	6	0.0904	LESS
24	72	40	5	0.0600	MORE
25	107	48	13	0.3574	LESS
26	50	20	8	0.1941	LESS
27	84	43	12	0.1947	MORE
28	18	14	5	0.0018	MORE
29	343	147	15	0.0640	LESS
30	551	254	18	0.3344	MORE
31	75	32	5	0.2573	MORE
32	551	266	27	0.2756	MORE
33	628	301	62	0.3238	MORE
34	334	172	25	0.0481	MORE
35	143	82	10	0.0056	MORE
36	31	20	6	0.0166	MORE
37	25	10	11	0.3062	LESS
39	93	46	13	0.2873	MORE
40	423	184	10	0.0754	LESS
41	60	30	5	0.2808	MORE
42	71	37	6	0.1672	MORE
44	32	17	8	0.1949	MORE
46	83	34	9	0.1562	LESS
47	104	40	23	0.0472	LESS
49	16	8	6	0.3140	LESS
50	37	21	6	0.0899	MORE
52	99	42	32	0.2031	LESS
53	41	14	5	0.0651	LESS

Limited to those 43 with probability less than 0.35 of 53 groups in total.

* Probability of this number (or more extreme comparisons) of SNP PAFs “Differing” out of the total, given an “a priori” probability of 0.471 for a “Differing” SNP PAF.

** MORE indicates an excess of PAFs differing between the populations and LESS means there was a relative deficit of PAFs differing between the populations.

The genes identified in the 7 groups with more or less than expected numbers of differing PAFs are listed in Table 3. Those groups with decreased numbers of differing PAFs included the genes for the collagen structural proteins, and genes coding for transmembrane proteins and the Kell blood group. Groups with increased numbers of differing PAFs included one with influence on glucose metabolism; one involved with immune function, and a group related to drug and metabolic detoxification pathways.

**Table 3:** Characteristics of 7 Gene Groups showing significantly more or less SNPs with differing prevalences.


Group 7 (collagen), p=0.0010 LESS					
COL6A2	COL4A5	COL9A2	COL4A2	COL4A1	COL4A6
Group 28 (insulin regulation, IGF1R), p=0.0018 MORE					
EIF2B1	EIF2B3	EIF2B4	EIF2B5	EIF2B2	
Group 35 (immune response, T-cell signaling), p=0.0056 MORE					
NFATC4	REL	TFAP2B	NFATC3	NFATC1	SOX5
NFAT5	NFATC2	FOXJ2	NFE2L2		
Group 21 (collagen), p=0.0156, LESS					
COL3A1	COL1A2	COL1A1	COL5A2	COL5A1	
Group 36 (dual specificity phosphatase, Erk/JNK pathway), p=0.0166, MORE					
DUSP9	DUSP7	DUSP10	DUSP5	DUSP6	DUSP2
Group 47 (transmembrane proteins, Kell blood group), p=0.0472, LESS					
TSPAN31	TMEM61	TMEM132E	TMEM63A	CD53	PRRT1
ARMC10	SMCR7	TMEM132D	MS4A6A	PDZK1IP1	C10orf72
MUSTN1	STARD3NL	ARV1	C12orf23	C14orf101	C14orf118
HAS3	EVC	XKR6	KIAA2013	CCDC109B	
Group 34 (cytochrome P450, drug metabolism), p=0.0481, MORE					
CYP4A11	CYP27A1	CYP2D6	CYP2E1	CYP2C9	TBXAS1
CYP3A4	CYP8B1	CYP7A1	CYP2J2	CYP4F2	CYP1A2
CYP2C19	CYP26A1	CYP7B1	CYP17A1	CYP2A6	CYP4B1
CYP3A5	CYP19A1	CYP1B1	CYP2A7	CYP2C18	CYP2C8

## Discussion

Population-based allelic prevalences are critical data for the integration and implementation of novel genetic findings into clinical and public health efforts, as well as furthering future genetic epidemiology investigations. The present report provides the most extensive data on PAFs within an American Indian population, both in terms of the very large number of SNPs genotyped (over 34,000) and the relatively large number of individuals genotyped (216). The value of this resource is enhanced by the fact that the basis of the identified ethnicity relies on official, legally maintained records dating back multiple generations, rather than self-reported ethnicity, as in most other studies.

For reasons that are unclear, there appears to be an increased number of monomorphic SNPs in the HapMap populations, as seen in Figure 1. The fact that over twice as many alleles were genotyped for the TMCC population creates an increased power to detect low allele frequencies and hence a decreased likelihood of monomorphic SNPs. It seems unlikely however, that this would produce the large difference between populations in proportion of SNPs that are monomorphic.

These data show a considerable degree of variation between majority and minority populations in overall SNP prevalence, even when comparing that portion of the American Indian population most closely related to Europeans. This is consistent with reports of differing (by arbitrary absolute values) PAFs of 22% [4] and 33% [5] between African American and Caucasian groups, and a somewhat smaller proportion (7.8%) comparing Caucasian and Mexican Americans. Garte et al. [14] have reported a similar degree of differing PAFs among two metabolic gene SNPs in Japan versus Korea and Singapore. An extensive genome-wide association study [15] of body mass index (BMI) among 1,120 Pima Indian participants, ascertained largely through familial relationships, used an over 900,000 SNP microarray but found a high rate (35%) of PAFs < 5%. In addition, one SNP with strong genome-wide association in the European population [16] and a PAF of 21% was monomorphic in this Pima population. This clearly illustrates the powerful effect of variant allele frequencies on both study design and population attributable risk.

Even in a very large Caucasian cohort with self-reported ancestral region of origin, there were still 19 of 51 SNPs (37%) with statistically differing PAFs [6]. The current analysis of three populations of self-identified Caucasian Europeans, showed a surprisingly high proportion (4/7, 57.1%) of pair-wise comparisons with differing PAFs.

It must be recognized, however, that the demonstration of statistically significant differences between populations using large sample sizes and very precise estimates does not translate into clinically (or public health related) important differences in PAF. Still, absolute differences in PAF of 10% and 20% between CEU and this American Indian cohort were found in 54.7% and 16.4% of SNPs, respectively. This provides cautionary information for those who may place undue confidence in the similarity of standard “ethnicities”, especially those which are self-reported.

When considering SNPs from functionally related genes, we see that 7 groups showed excessive or reduced numbers of differing PAFs and that this distribution of groups with a p value less than 0.05 is highly unusual (p=0.0046). The presence of fewer differing PAFs could be due to selection pressure since two of the three groups coded for collagen, which may well be under selection constraints. The groups with more differing PAFs could be explained by genes with minimal selection pressure or a founder effect.

The functionality of those groups with fewer differing PAFs seemed more related to basic physiologic mechanisms, such as the structural collagen genes and certain of the transmembrane proteins, possibly involved in signaling. This naturally suggests that these critical functions may be more affected by selection pressure at the species level, and require more uniformity and representation of these SNPs. Those with more differing PAFs involved immune response, detoxification metabolism and glucose metabolism. The latter is of particular interest given the increased prevalence of diabetes in many American Indian populations [17].

This study would clearly be strengthened if a greater number of genotypes were available for both the American Indian and HapMap PAFs, but the countervailing strength of both these sources is the very large number of SNPs available for analysis (tens of thousands) in contrast to the only other analyses with thousands of genotypes on tens of SNPs. The other strength of the present report is the use of objective, carefully maintained, records of ethnic origin, rather than self-reported ethnicity. The only other report of population-based American Indian PAFs genotyped only 51 SNPs among 167 individuals of self-reported ethnicity [6].

In conclusion, these results from a very large number of SNPs genotyped in a substantial number of American Indian participants, ascertained in a population-based manner show that a high proportion of SNP population allele frequencies differ from those reported in Caucasians from the HapMap dataset. There also appear to be groups of genes with certain functionalities which have significantly more or less differing PAFs. These results will be useful in the design of future genetic epidemiology studies and may eventually find utility in public health or clinical screening efforts.

## Supporting Information

Figure S1 Plot of Principal Components 1 vs 2, showing dichotomized TMCC population and selected comparison populations. (TIF)
