# Supplementary figures and images for: IBC CARe Microarray Allelic Population Prevalences in an American Indian Population
Source: PLoS One. 2013 Sep 6;8(9):e75080. doi: 10.1371/journal.pone.0075080 (PMC3765406; doi:10.1371/journal.pone.0075080)

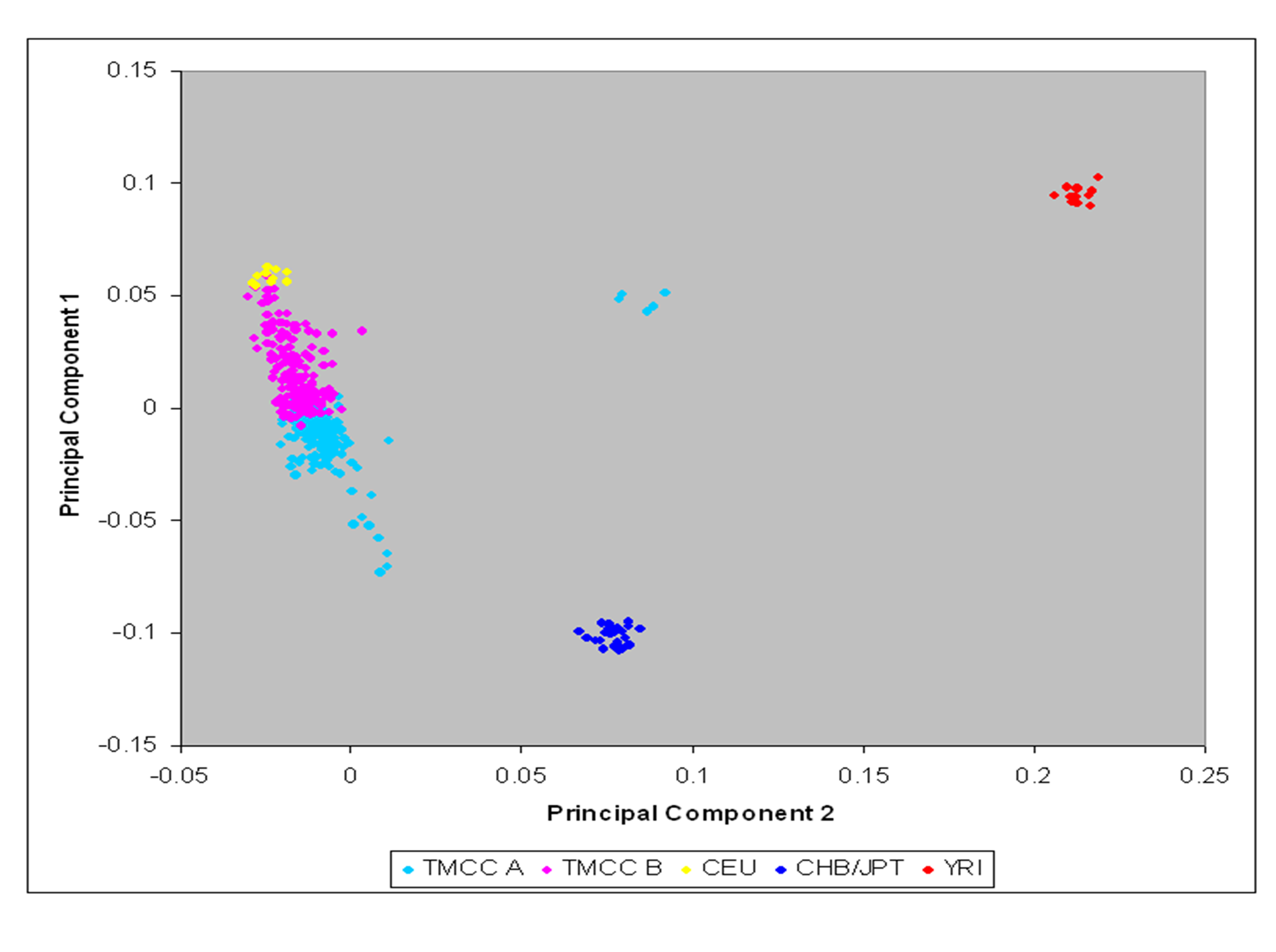

Supplement: Figure S1 — (TIF) [file pone.0075080.s001.tif]
